# Novel Interventions to Improve Adherence to Guideline-Directed Medical Therapy in Claudicants

**DOI:** 10.3390/jcm14155309

**Published:** 2025-07-28

**Authors:** Richard Shi, Nicholas Bulatao, Adam Tanious

**Affiliations:** Department of Vascular Surgery, Medical University of South Carolina, Charleston, SC 29425, USA; nicholas.bulatao@live.mercer.edu (N.B.); tanious@musc.edu (A.T.)

**Keywords:** peripheral arterial disease, claudication, quality improvement, guideline-directed medical therapy, medication adherence

## Abstract

Intermittent claudication is the most common manifestation of peripheral arterial disease as well as a lifestyle-limiting disease with a favorable prognosis. Despite societal guideline recommendations, most claudicants do not trial optimal medical therapy (OMT) and supervised exercise therapy (SET) or receive a quality-of-life (QoL) assessment prior to intervention. In this review, we discuss the components of OMT and SET and the trials establishing their clear benefits in claudicants. We assess adherence rates to OMT/SET and qualitative and quantitative studies attempting to understand the barriers to adoption. We also review how patient-reported outcome metrics were developed to assess QoL in claudicants and reasons for their underutilization in daily clinical practice. Last, we describe novel initiatives seeking to improve adherence to OMT, SET, and QoL assessment.

## 1. Introduction

Peripheral arterial disease (PAD) affects more than 230 million individuals globally [1]. Its most common manifestation is intermittent claudication, defined as exertion-induced muscle pain of the lower extremities resolved with rest [2].

The prognosis of IC is favorable. Over two-thirds of claudicants will have symptoms that remain stable or improve without intervention. Furthermore, the rate of limb loss is less than 1% per year [3]. Despite this, intervention rates for claudication have risen over the past decade [4]. There is no clinical evidence suggesting that surgical intervention improves walking distance and quality of life (QoL) compared with non-surgical options like supervised exercise therapy (SET) and optimal medical therapy (OMT) [5,6]. Premature surgical intervention is also a predictor for progression to CLTI [7].

Subsequently, multiple surgical societies have published guidelines stating that intervention for claudication should be reserved for patients with severe lifestyle-limiting symptoms that have failed OMT and SET [3,8,9]. Adherence to such guidelines lacks consistency across healthcare systems [10]. An analysis of the Vascular Quality Initiative (VQI) data found that only one-third of claudicants who underwent elective revascularization were medically optimized prior to intervention [11]. Additionally, a US-based survey found that almost half of vascular specialists had never referred a patient to an SET program [12]. Additionally, it is unclear the degree to which surgeons assess the severity of lifestyle limitation and quality of life (QoL) in claudicants. Patient-reported outcome measures (PROMs), such as the Vascular Quality of Life Questionnaire (VascuQoL) or the Walking Impairment Questionnaire (WIQ), are gold standards in measuring QoL changes but are not yet widely utilized clinically.

Adherence to guideline-directed medical therapy (GDMT) and reporting of lifestyle limitation/QoL remains sub-optimal. This article provides a contemporary review on the benefits of OMT, SET, and PROMs usage, qualitative/quantitative analyses exploring their adherence, and innovative quality improvement interventions seeking to improve their utilization.

## 2. Optimal Medical Therapy

### 2.1. Current Guideline Recommendations

**Figure 1:**
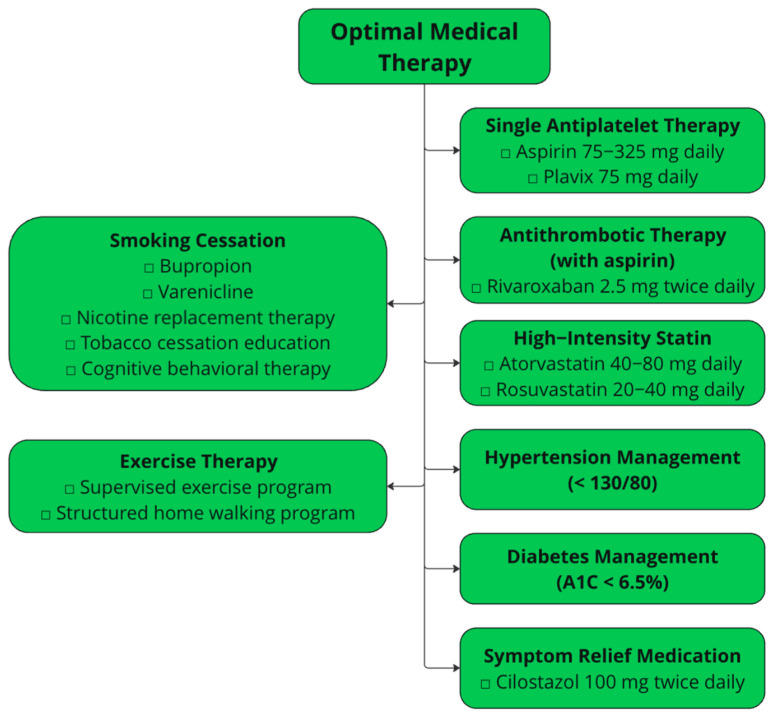
The tenets of optimal medical therapy per the ACC/AHA Guideline for the Management of Lower Extremity Peripheral Arterial Disease.

OMT is the cornerstone of non-surgical management for claudication and reduces disease progression, major adverse limb events (MALEs), and major adverse cardiac events (MACEs). OMT, compared with surgical intervention, also demonstrates greater improvements in patient-reported outcome measures [13]. Per the ACC/AHA Guideline for the Management of Lower Extremity Peripheral Arterial Disease, OMT involves antiplatelet and antithrombotic therapy, lipid-lowering therapy, smoking cessation, antihypertensive therapy, diabetes management, and symptom management therapy (Figure 1) [9].

Antiplatelet therapy involves a single antiplatelet agent, typically aspirin alone (75–325 mg daily) or clopidogrel (75 mg daily). Antiplatelet therapy provides cardioprotective benefits, as it reduces the risk of MACEs in patients with symptomatic PAD [14,15]. Dual antiplatelet therapy in patients prior to revascularization is not recommended due to higher rates of bleeding.

Antithrombotic therapy refers to low-dose rivaroxaban (2.5 mg twice daily) in conjunction with single antiplatelet therapy. These recommendations are based on the COMPASS and VOYAGER randomized clinical trials, which showed improved cardiovascular outcomes and MALEs in patients with PAD taking both ASA and rivaroxaban compared with ASA alone [16,17,18]. A sub-group analysis of the COMPASS trial also investigated the impact of rivaroxaban 2.5 mg plus aspirin versus rivaroxaban 5 mg alone and aspirin alone in patients with PAD. This analysis revealed that the primary outcome (composite of cardiovascular death, myocardial infarction, and stroke) was significantly reduced in the rivaroxaban plus aspirin group versus the aspirin alone group. No differences were seen in the rivaroxaban alone group compared with the aspirin alone group [19]. A sub-analysis from the VOYAGER PAD trial investigated whether the addition of clopidogrel impacted the effects of rivaroxaban plus aspirin in patients with revascularization for PAD. This study revealed that the addition of clopidogrel did not change the efficacy outcome of acute limb ischemia, major amputation, myocardial infarction, ischemia stroke, and cardiovascular death. However, there was a trend toward increased bleeding risk if clopidogrel was used over 30 days [20].

Lipid-lowering therapies include treatment with high-intensity statins, such as atorvastatin 40–80 mg or rosuvastatin 20–40 mg, with the goal of reducing low-density lipoprotein cholesterol (LDL-C) by >50%. Both RCTs and large database studies have observed decreases in MACEs, MALEs, and death rates in patients with PAD on statin therapies. In patients maximally tolerated on statin therapy with LDL-C levels greater than 70 mg/dL, or those unable to tolerate statins, other options include ezetimibe and PCSK9-inhibitor therapy. Multiple RCTs demonstrate the benefit of alirocumab and evolocumab in lowering rates of MACEs and MALEs compared with placebo in patients with PAD [21,22]. For ezetimibe, an RCT of patients with extracoronary atherosclerotic arterial disease showed that patients with the addition of ezetimibe to simvastatin had reduced MACEs [23]. No studies have shown the impact of ezetimibe on MALEs.

Smoking cessation, including exposure to secondhand tobacco smoke, is recommended in all patients with PAD. Smoking cessation has been shown to reduce MALEs, particularly bypass failures and amputations [24,25]. Some smoking cessation methods include pharmacotherapy such as varenicline, bupropion, and nicotine patches and behavioral therapies such as cognitive behavioral therapy and motivational interviewing [26].

Antihypertensive therapy is recommended in claudicants with a systolic blood pressure goal of <130 mmHg and diastolic blood pressure goal of <80 mmHg. Blood pressure control reduces the risk of stroke, cardiovascular death, and even MALEs, especially for angiotensin-converting enzyme (ACE) inhibitors or angiotensin-receptor blockers (ARBs) [27,28,29].

Diabetes management, notably with glucagon-like peptide-1 (GLP-1) agonists and sodium–glucose transport protein 2 (SGLT2) inhibitors, minimizes the MACEs in claudicants [30,31]. No hemoglobin A1C value is recommended, although studies have investigated outcomes with values below 6.5% [32].

Medications to manage leg symptoms include cilostazol, a phosphodiesterase III (PDE3) inhibitor causing vasodilation and platelet aggregation inhibition. Cilostazol improves leg symptoms, quality of life, and walking distance, as shown in multiple RCTs [33,34,35]. Cilostazol is contraindicated in patients with congestive heart failure since it is a PDE3 inhibitor, which is known to worsen mortality rates in this population [36]. Pentoxifylline is another medical therapy used historically to improve pain-free walking distance. While well tolerated, the literature investigating pentoxifylline in claudicants is highly variable, and the assessment of effectiveness is uncertain [37]. An RCT of 698 patients compared mean maximal walking distance in those treated with cilostazol, pentoxifylline, or placebo. At 24 weeks, improvement in maximal walking distance was significantly higher in the cilostazol group compared with the pentoxifylline and control group [38]. As a result, pentoxifylline is not currently recommended by the ACC and AHA for the treatment of claudication [9]. Similarly, prostaglandins, which have vasodilatory and antiplatelet formation effects, have not been shown to improve walking distance or quality of life in claudicants compared with cilostazol. There remains insufficient evidence investigating the impact of prostaglandins on intermittent claudication, and thus, remains unrecommended by claudication guidelines [39].

According to the ACA/AHA guidelines, healthy nutrition is vital for preventing the development of atherosclerotic cardiovascular disease and reducing MACEs. The recommended diet consists of vegetables, fruits, legumes, nuts, whole grains, and fish. This is supported by the PREDIMED (Prevención con Dieta Mediterránea) trial, which found that a Mediterranean diet supplemented with extra-virgin olive oil and mixed nuts had a reduced number of cardiovascular events compared with a standard reduced-fat diet [40].

### 2.2. Adherence Rates to OMT

Adherence rates to OMT are varied, but in general are low. A study of 39,088 claudicants with surgical intervention in the Vascular Quality Initiative (VQI) revealed that only 38.1% were on complete OMT, defined as antiplatelet, statin, and smoking cessation. Furthermore, 54.6% were on partial OMT, defined as meeting 1–2 components. Both complete and partial OMT decreased the odds of reintervention and MALEs compared with no OMT. For individual components, 76.3% were on an aspirin, 76.2% were on a statin, and 53.4% demonstrated smoking cessation [11]. Similarly, the PORTRAIT (Patient-Centered Outcomes Related to Treatment Practices in Peripheral Arterial Disease) registry investigated adherence to guideline-recommended therapy in 1275 patients with PAD across 16 international centers from 2011 to 2015. They documented four measures: antiplatelets, statins, smoking cessation counseling/therapy, and SET referral. A total of 77.2% were adherent to two quality measures, while 19.7% were adherent to four quality measures [41]. Furthermore, only a third of smokers with PAD were ever offered smoking cessation counseling or pharmacologic treatment [42].

For cilostazol, in a VQI study of 245,309 patients undergoing peripheral vascular intervention for claudication or CLTI, only 6.6% of patients were on cilostazol prior to intervention. Patients on cilostazol ultimately had lower rates of long-term mortality and major amputation [43]. In terms of antihypertensive regiments, a UK-based cohort study found prescription rates of ACE inhibitors/ARBs to be above 40% in patients two months after a diagnosis of PAD [44]. For diabetes, a VQI study of diabetics with lower extremity bypass demonstrated that 25% had no documented A1C, 29% had a documented A1C of <7%, and 46% had an A1C of above 7% [45]. These results reveal that many patients are not medically optimized prior to undergoing surgical intervention.

There are qualitative studies attempting to understand patient factors in low medication adherence. Lampridou et al. explored patient themes regarding medication adherence. One theme was the lack of perceived symptom benefits with GDMT, as its dominant impact is its cardioprotective and limb-protective effects rather than symptom relief or functional improvement. Another theme was lack of clarity regarding the purpose of the numerous medications and the fear of polypharmacy. Certain patients also complained about the side effects of certain medications, such as muscle pain from statin therapy or bleeding from antiplatelets/anticoagulation. Lastly, adequate social support from family/friends and partnering with their healthcare provider appeared to be a key factor in maintaining medication adherence [46].

### 2.3. Current and Upcoming Interventions

Multiple qualitative improvement initiatives aiming to improve GDMT adherence have been published, with varying levels of success (Table 1). Haile et al. developed a nursing-led follow-up program to maintain adherence to lipid-lowering medications, antiplatelets, and anticoagulants in claudicants following intervention. The intervention involved three nursing visits and two telephone calls by a vascular nurse in the first year after revascularization. Their RCT revealed no difference in medication adherence between the intervention and control group. They noted their baseline adherence rates were high, making it difficult to achieve any significant improvements [47]. Another RCT assessed the ability of medication counseling via telephone calls to improve medication adherence among 1446 participants with abdominal aortic aneurysms, peripheral arterial disease, or hypertension. Their primary outcome was composite adherence to statin, antithrombotic, and antihypertensive treatment. While an increase in statin prescriptions was seen at 6 months, there was no change in adherence among the composite of three medications at 12 and 60 months of follow-up [48].

Mobile health interventions have been developed to modify patient behavior. A multicenter RCT of 822 patients investigated the impact of 6 text messages a week for 6 months regarding motivational content and education related to disease knowledge, risk factor control, and medication adherence for hypertension. Although patients found the messages useful, there was no change in systolic blood pressure, cholesterol level, or physical activity between the intervention and control groups [49]. Similarly, another RCT found that weekly text messages for 6 months regarding risk factor modification in patients with percutaneous coronary interventions increased the level of physical activity, fruit/vegetable consumption, and medication adherence. Despite this, no changes in blood pressure, cholesterol levels, or body-mass index were seen [50].

Virani et al. utilized a natural language processing algorithm to analyze clinical documentation and identify reasons for non-adherence to high-intensity statins in patients with cardiovascular disease. Medication side effects were the largest barrier. Thus, a clinical decision support tool was designed that alerted physicians before clinic visits whether a patient needed a statin, any existing side effects, and guideline resources [51]. Baseline adherence rates of high-intensity statins at the intervention clinics (*n* = 14) were 53.6%, and they increased by 10.1% post-intervention. In the control clinic (*n* = 13), the baseline rate was 55.9%, and this decreased by 0.18% post-intervention [52]. This study reveals the impact of developing interventions based on identified root issues.

Beyond electronic-based interventions, innovations in drug delivery may also impact adherence. Castellano et al. investigated the impact of a polypill containing ASA 100 mg, simvastatin 40 mg, and ramipril 2.5/5/10 mg on medication adherence for secondary prevention post-myocardial infarction, compared with the three drugs given separately. They performed an RCT with a five-country cohort of 2118 patients and found that the polypill group (50.8%) demonstrated improved adherence after 9 months from baseline (41.0%) [53].

Improving medication adherence remains a difficult challenge, as many interventions are unable to achieve sustained patient benefits. These likely reflect the difficulties in designing an effective intervention and changing patient behaviors. Ultimately, promising interventions will emphasize understanding the root causes of medication non-adherence and focus on improving the partnership between the provider and patient to achieve improved care within claudicants.

**Table 1:** Interventions to improve adherence to optimal medical therapy.

Study Title and Year	Intervention	Control	Study Sample	Primary Outcome	Results
Effects of a person−centered, nurse−led follow−up programme on adherence to prescribed medication among patients surgically treated for intermittent claudication: randomized clinical trial (2022) [47]	Three visits and two telephone calls by a specially trained vascular nurse over one year after revascularization	Two visits after surgery to vascular surgeon or nurse	Two centers, 214 patients	Proportion of days covered (PDC) (number of available dispensed doses via registry data/number of days patient prescribed the medication)	No difference in PDC for lipid−modifying agents, antiplatelets, anticoagulants
Randomised trial of telephone counselling to improve participants' adherence to prescribed drugs in a vascular screening trial (2020) [48]	5−15 min sessions of telephone counseling by nurse regarding refilling prescriptions, side effects, and health advice	No telephone counseling	1446 patients with AAA, PAD, or hypertension	Proportion of days covered (PDC) by statin, antithrombotic, and antihypertensive agents at 6−month follow-up	Increase in PDC for statins at 6 months. No other differences noted at 6, 12, and 60 months.
Effect of Text Messaging on Risk Factor Management in Patients With Coronary Heart Disease: The CHAT Randomized Clinical Trial (2019) [49]	Six texts/week of education information related to disease knowledge, physical activity, and medication adherence	Two thank you text messages a month	37 hospitals, 822 patients with coronary heart disease	Systolic blood pressure at six months	No differences in blood pressure at six months. No differences in LDL, physical activity, or smoking activity.
Cluster Randomized Trial of a Personalized Clinical Decision Support Intervention to Improve Statin Prescribing in Patients With Atherosclerotic Cardiovascular Disease (2023) [52]	Clinical decision support tool to alerts physicians before clinic visits about current statin and dose, date of last fill, type of side effect	Usual clinician access to patient dashboard detailing compliance with statin	27 primary care clinics, 36,641 patients with cardiovascular disease	Statin adherence (defined as proportion of days covered for statin > 80%)	Absolute change in statin adherence was +10.1% in intervention group vs −0.18% decrease in control group
A polypill strategy to improve adherence: results from the FOCUS project (2014) [53]	Polypill containing aspirin 100 mg, simvastatin 40 mg, and ramipril 2.5/5/10 mg	Three drugs given separately	2118 patients	Medication adherence via Morisky−Green Questionnaire (MAQ) and pill count after nine months	Adherence was 50.8% in intervention group versus 41% (*p* = 0.02). No difference in blood pressure or cholesterol levels.

Alongside OMT, supervised exercise therapy (SET) is recommended as a first-line intervention in claudicants prior to surgical revascularization. SET involves walking on a treadmill at a speed that induces moderate claudication pain within several minutes, guided by a physical therapist. The patient stops and rests until the pain improves, upon which they will resume walking. This cycle is repeated for at least 30 min to 1 hour and should be performed at least three times a week. The Centers for Medicare and Medicaid Services currently covers up to 36 separate 30–60 min sessions over a 12-week treatment period [54].

## 3. Supervised Exercise Therapy

### 3.1. Mechanisms and Benefits of Supervised Exercise Therapy

There are several mechanisms by which SET provides patient benefit. SET causes exercise-induced angiogenesis, creating collateral blood supply around atherosclerotic occlusions. SET also causes nitric oxide-dependent vasodilation of the arterial microcirculation, improving perfusion. Studies have also shown that the metabolism of glucose and fatty acids by skeletal myocytes is improved during SET. These mechanisms all improve functional outcomes within claudicants.

Multiple RCTs showcase the durable benefits of SET. In the CLEVER (Claudication: Exercise Versus Endoluminal Revascularization) study by Murphy et al., SET and OMT showed comparable improvements in claudication-onset time and peak walking time to surgical revascularization at 18 months post-intervention [55]. Fakhry et al. performed an RCT of 212 claudicants assessing the effectiveness of endovascular revascularization with SET versus SET alone. At the one-year follow-up, combination therapy demonstrated greater improvements in maximum walking distance, pain-free walking distance, VascuQol scores, and the SF-36 physical functioning scores [56].

### 3.2. Adherence to Supervised Exercise Therapy

Despite the benefits of SET, its utilization rate in real-world practice is poor. Published rates of SET completion range anywhere between 5% and 55% [57]. A systematic review of 23 RCTs for SET found that of 7517 screened total patients, only 24.2% were recruited. Of those recruited, only 75% adequately completed the program. The top reasons for screen failure included lack of interest in SET, comorbidities affecting the ability to exercise, and the inability to attend SET due to distance/timing. The top reasons for incomplete adherence were a lack of motivation, comorbidities, lack of results, and patient choice [58]. Likewise, in a questionnaire of 516 patients with PAD, 85.1% revealed their physician never prescribed or recommended SET, despite 73.2% willing to travel several times a week to participate. However, only 41% were willing to pay the $11 per session copay. Patients unwilling to travel to SET felt it was too time-consuming, too inconvenient, and overall lacked interest [59]. Similarly, in a survey of 135 vascular surgeons, vascular medicine physicians, and cardiologists, 54% had no SET program at their facility, 49% had never referred a patient to SET, and 26% were unaware that SET sessions were covered by CMS. These are daunting statistics showcasing a lack of fundamental knowledge of SET even among vascular specialists [12]. Despite being covered by Medicaid, SET adherence remains greatly impacted by the lack of awareness around SET, interference with daily life due to session frequency/timing, and lack of motivation to attend dozens of sessions.

### 3.3. Interventions to Enhance and Facilitate SET

Recent initiatives have attempted to address exercise therapy adherence (Table 2). One strategy is performing exercise therapy at home through structured, community-based walking programs. In these programs, patients follow a guided program with the aid of a virtual coach/physical therapist. The Group Oriented Arterial Leg Study (GOALS) compared outcomes of a home-based group-mediated cognitive behavioral walking intervention versus a control group exposed to general health education. The experimental group received six months of PAD- and SET-specific weekly group education from a trained facilitator as well as a paper form to track weekly walking goals and distance. Compared with the control, patients within the intervention group reported significant increases in six-minute walking distance, maximum treadmill walking distance, and walking impairment questionnaire distance and speed score [60]. The LITE RCT investigated the effectiveness of various intensities of a home-based walking program. They compared a low-intensity program where ischemic symptoms were not induced to a high-intensity group that walked at a pace to induce moderate to severe ischemic leg symptoms. The high-intensity walking group demonstrated a higher six-minute walk distance at 12 months [61]. These studies indicate the effectiveness of allowing patients to walk at their convenience without the need to travel. However, a facilitator is required to provide motivation and ensure patients are walking at a sufficient intensity.

Mobile health interventions have been an increasingly popular method to deliver and facilitate walking programs. Normahani et al. conducted an RCT investigating the impact of a wearable activity monitor (WAM) to assist the delivery of a home-based walking program. The intervention group received 3 months of SET followed by 9 months of home walking program monitored by a WAM; the control group received only SET. At one year, there were higher improvements in mean walking distance and VascuQoL score in the intervention group compared with the control [62]. Paldan et al. developed a smartphone app, TrackPAD, to record training duration/frequency, step count, and pain levels during SET. In addition, the app sets weekly goals and “gamifies” the experience through achievement medals and leaderboards to compete with other users. In their RCT, the intervention group increased their six-minute walking distance at three months, with the control group demonstrating no improvement [63]. Mobile health programs are an effective way to deliver personalized coaching and track walking progress, especially with the ubiquity of smartphones and wearable devices.

Financial incentives can also be a creative way to tackle SET adherence. Cleary et al. utilized a three-pronged method involving financial incentives up to $180, individualized coaching, and informational materials to improve their SET adherence rate from 54% to 76.7% over a two-year period. This study revealed that the hidden associated costs of SET (copays, transportation costs, unpaid leave from work) are underrecognized barriers to adherence [64].

In summary, SET adherence remains a difficult challenge. Successful interventions include health coaching and physician partnership to provide continuous education and maintain accountability. SET access is also a major issue; SET facilities need to be expanded, and access to quality home walking programs need to be provided to patients. Ultimately, the future of exercise therapy may involve home-based programs delivered through mobile apps and financial incentives to offset the hidden costs of SET.

An important metric when providing guideline-directed care for claudicants is the assessment of the quality of life and the degree of lifestyle limitation. Given that claudication is primarily a lifestyle disease, there has been increasing interest in utilizing patient-reported outcome measures (PROMs) to assess the functional outcomes of surgical intervention. Despite a host of validated surveys and recommendations from the AHA/ACC guidelines recommending periodic assessment of health-related quality of life, PROMs remain underutilized.

## 4. Patient-Reported Outcome Measures 

### 4.1. Existing PROMs for Claudication

According to the FDA, PROMs are tools that serve as a report of the status of a patient’s health condition, health behavior, or experience with healthcare that comes directly from the patient, without interpretation by a clinician or anyone else [65]. PROMs are frequently utilized in comparative effectiveness research and to monitor disease states. PROMs feature questions that represent a “domain” of assessment, such as pain, psychological impact, impact on social activities, and impact on daily living. These domains will be scored to present an overall assessment of the patient’s quality of life (QoL). PROMs are developed through a conceptual framework inspired by the existing literature and focus groups/patient interviews. Once a draft instrument is developed, PROMs are repeatedly tested among hundreds of patients to ensure survey validity, reliability, and responsiveness. PROMs also undergo translation and cultural adaptation so they can be utilized among patients from diverse socioeconomic and health backgrounds [66].

Most randomized clinical trials regarding peripheral arterial disease utilize one generic and disease-specific PROM to assess QoL and surgical outcomes. The most commonly used generic PROMs include the 36-Item Short Form Survey (SF-36) and the European Quality of Life 5-Dimension (EQ-5D). The SF-36, developed in 1992, is a survey of 36 questions assessing 8 health domains: (1) limitations in physical activities, (2) limitations in social activities, (3) limitations in usual role activities, (4) bodily pain, (5) general mental health, (6) limitations in usual role activities due to emotional problems, (7) vitality, and (8) general health perceptions [67]. A score from 0 to 100 is counted, with 100 being the highest possible score. Studies have found that patients with claudication report ultimately lower scores in the physical functioning/limitation components of the SF-36, as well as bodily pain, when compared with healthy controls [68]. The EQ-5D is a generic PROM developed by the EuroQol Group, aiming to measure five dimensions of health states: mobility, usual activities, self-care, pain and discomfort, and anxiety and depression. Compared with the SF-36, the EQ-5D is shorter and can generate utility scores for use in cost analysis research [69].

Both the SF-36 and EQ-5D have been widely utilized to examine quality of life outcomes in several major clinical trials for PAD. For example, the ERASE clinical trial compared the effectiveness of endovascular surgery with SET versus SET alone in 212 patients. They found that the combination therapy demonstrated a higher SF-36 score in the physical functioning domain but not in the physical role functioning, bodily pain, and general health perception [70]. The Disrupt PAD III RCT compared the use of intravascular lithotripsy versus balloon angioplasty in severely calcified femoropopliteal arteries prior to stenting or drug-coated balloon treatment. EQ-5D values were a secondary outcome in this study, and results showed no differences in the increase from baseline at one year, indicating a similar quality of life improvement between the two interventions [71].

**Figure 2:**
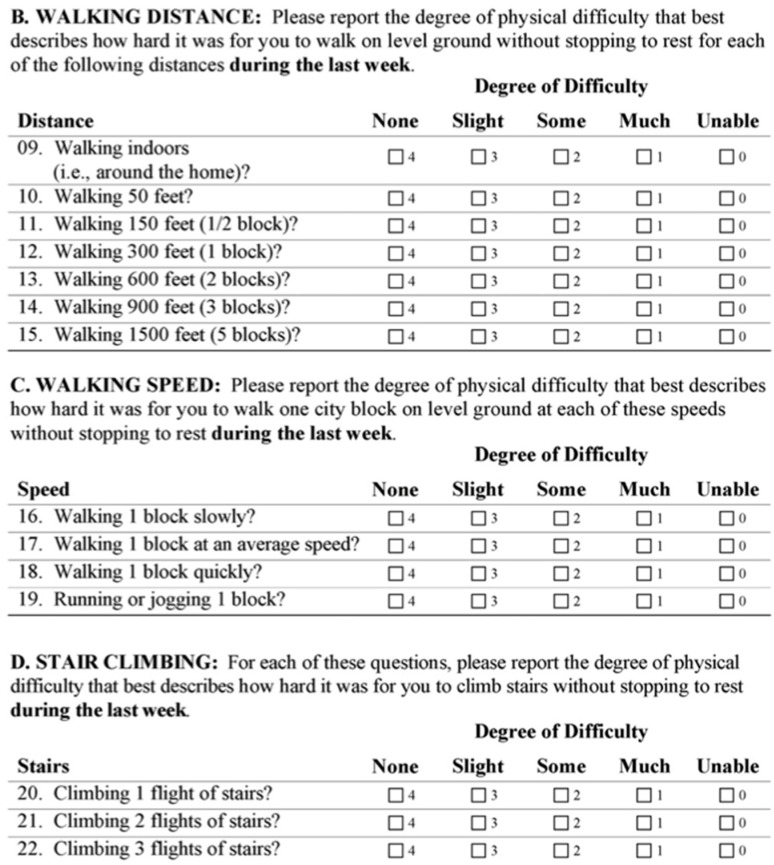
Walking distance and stair climbing component of the Walking Impairment Questionnaire [73].

One of the most popular PROMs designed specifically for PAD is the Vascular Quality of Life Questionnaire (VascuQoL). The VascuQol is a 25-question survey assessing 5 quality-of-life domains: pain, symptoms, activities, social impact, and emotional impact. The survey is designed to take 9 min to answer, and its shorter version, the VascuQoL-6, is a six-question, shortened version designed to take 1.4 min. The VascuQoL is a popular PROM for PAD research. Compared with the SF-37 and EQ-5D, the VascuQoL-6 has been shown to have a higher receiver operating characteristic area under the curve when detecting improvements in Rutherford classification at six-month follow-up in a prospective study of 450 patients with PAD [72]. Compared with the VascuQoL, the Walking Impairment Questionnaire (WIQ) is a more specific measurement of functional status and directly assesses the walking ability of patients. The WIQ is a 22-question survey designed to measure self-reported walking distance, walking speed, and stair-climbing limitation (Figure 2). The WIQ has been validated in patients with claudication and was shown to be highly correlated with the six-minute walk and measured walking velocity in both men and women [73]. In fact, the WIQ has even been shown to predict mortality, as a study by Jain et al. revealed that patients with PAD in the lowest quartile of WIQ stair-climbing scores had higher all-cause and cardiovascular mortality than those in the highest quartile [74].

### 4.2. Barriers to PROM Implementation

The validation and utility of PROMs for assessing outcomes research for PAD and claudication is evident. However, its adoption in real-world clinical practice is low, given that these outcome measures have been used primarily in research settings. There is an ongoing effort by the Society of Vascular Surgery to improve the implementation of PROMs in the community.

In 2021, the SVS distributed a survey to 106 SVS members serving on the Policy and Advocacy Council, with 78 respondents ultimately. They found that while 80.8% were aware of PROMs, and 100% felt they would be useful in assessing vascular surgery patients, only 23.1% indicated that their practice or institution used PROMs. Of those utilizing PROMs, the most commonly cited reasons for using them were research/quality improvement initiatives followed by institutional requirements and quality reporting. Furthermore, 70% of respondents indicated they would consider using PROMs if incorporated into electronic medical records. Reasons for not collecting PROMs included existing PROMs not being specific to the patient’s problem, difficulty obtaining results, or inability to analyze the collected data [66].

This survey was followed by a one-hour focus group among respondents. One major theme around PROMs was knowledge gaps. Specifically, there was lack of awareness of all the various types of PROM and how PROMs are developed and validated. Many clinicians were unsure of how to utilize PROMs in their practice and how they should guide patient care. It was also unclear which ones to use, as surgical societies do not currently recommend a specific PROM. Another theme was the actual implementation barriers of PROMs. RCTs have the dedicated staff and resources to collect PROM data and input them into database registries, which are not typically performed in daily clinical practice. While electronic methods could be a good alternative, it was felt that the vascular surgery population, which is primarily elderly, may be limited in their ability to respond. Another concern was how PROM results would be interpreted by payers and policy makers and how this may ultimately affect reimbursement [66].

Beyond physician perception, patients themselves face burdens when filling out PROMs. Holeman et al. performed a study investigating patient perceptions of PROMs through interviewing 23 patients with PAD. In terms of PROM timing, patients prefer to receive them prior to the appointment through email or text. Some patients also felt the PROM questions were not specific enough to their direct symptoms or overall health. Despite this, patients felt that PROMs improved communication with their providers and felt they helped track progress over time. Lastly, only 65% of the patients felt that the PROMs were valuable. Those that disagreed with its value felt that the questions were repetitive and focused on previously discussed information with the provider. Others questioned their utility to the surgeon [75].

### 4.3. Keys to PROM Adoption

Efforts to improve the adoption rate of PROMs need to accelerate, especially as Medicare reimbursement for certain elective surgical procedures will begin to require collection of PROMs as early as 2026 [76]. Improving the adoption of PROMs will require consideration of patient and clinician factors.

First, there should be a standardized, singular PROM that caters to the claudication patient’s symptoms and concern. In a systematic review of 31 studies that recorded PROM usage for claudicants, there were 14 different questionnaires. The most common were the SF-36, EQ-5D, VascuQoL, and WIQ [77]. While these individually assess different aspects of claudication, it is challenging to implement all these PROMs clinically. Surgical societies should provide guidance on which PROMs should be used to help standardize the process of recording QoL metrics.

Second, the designs of the PROMs need to be intuitive and cannot create significant patient and clinician burden to fill out. Several existing PROMs take over 10 min to fill out [66]. Studies have shown that PROM response rates tend to be higher when questionnaires are shorter [78]. Thus, there needs to be a balance between PROM comprehensiveness and efficiency. Furthermore, in the setting of shortened appointment times, PROMs should be delivered to patients prior to appointments via email/smartphone at home. If performed at the clinic, patients should be provided with sufficient time to complete the PROMs prior to seeing the physician as well as with devices that may facilitate the ease of data collection, such as iPads. Qualitative studies have found that electronic PROMs can minimize responder burden and improve compliance [58,79]. Electronic PROMs can also feature additional functionalities to facilitate completion. For example, using the concept of computerized adaptive testing, PROMs can become dynamic, where certain questions only appear depending on the answer to the previous one, preventing the patient from answering irrelevant questions [80].

Third, there needs to be increasing clarity in when and how PROMs for claudicants should be used, for both the patient and physician. Patient education should be provided regarding what PROMs are used for and how certain scores may correlate with certain surgical outcomes. Furthermore, giving patients and physicians the ability to track their PROMs over time through numerical and visual graphics can increase engagement and response compliance [81,82]. This allows both parties to understand the impact of the existing disease and the impact of medical/surgical interventions on overall QoL. On the physician end, there should be further education on the minimal clinically meaningful difference (MCID) for each PROM to help them understand the minimum change in scores that patients would consider beneficial. MCIDs are different for each PROM, and furthermore, vary depending on the patient’s baseline demographics and disease severity [83]. Without known MCIDs, physicians cannot correlate PROMs with patient outcomes, minimizing their usefulness.

## 5. Conclusions

Despite their obvious benefits for claudicants, adherence to OMT, SET, and QoL assessment remains an extremely difficult challenge. While numerous RCTs and qualitative/quantitative studies have sought to address these issues, many are unsuccessful due to patient behaviors or perceptions not being directly addressed. Successful interventions will involve a root-cause analysis of the existing patient challenges to adherence and recurring patient education and health coaching. Future research should continue to emphasize exciting and novel approaches to OMT, SET, and QoL assessment adherence.

## Figures and Tables

**Table 2:** Novel interventions to enhance supervised exercise therapy adherence.

Study Title and Year	Intervention	Control	Study Sample	Primary Outcome	Results
Home−Based Walking Exercise Intervention in Peripheral Artery Disease A Randomized Clinical Trial (2013) [60]	Home−based group−mediated cognitive behavioral walking intervention	Weekly lectures on health−related topics	194 patients	Six−minute walk performance at six−month follow-up	Intervention group increased 6−minute walk distance from 357.4 to 399.8 meters. Control group decreased from 353.3 to 342.2 m.
Wearable Sensor Technology Efficacy in Peripheral Vascular Disease (wSTEP): A Randomized Controlled Trial (2018) [62]	Wrist-worn activity monitor to track and encourage daily walking, in addition to SET session	SET only	37 patients	Maximum walking distance at 3, 6, and 12 months	Intervention group showed improvement in MWD at 3, 6, and 12 months. No improvements were seen in control group.
Supervised Exercise Therapy Using Mobile Health Technology in Patients With Peripheral Arterial Disease: Pilot Randomized Controlled Trial (2021) [63]	TrackPAD app to track SET sessions. App monitors training duration, step count, and pain levels.	SET only	39 patients	Six−minute walking distance at three months	Intervention group increased their six−minute walking distance. Control group decreased their walking distance.
Incentives and individualized coaching improve completion rates of supervised exercise therapy for claudication (2024) [64]	Financial incentive of $180, scheduled health coaching, information materials, alongside SET	SET only	73 patients	SET completion rate	Intervention increased SET completion rate from 54% to 76.7%

## Data Availability

No new data was created or analyzed in this study.
